# Development of the acne-specific quality of life questionnaire Quality of Life Relevance-Acne

**DOI:** 10.1016/j.jdin.2024.03.007

**Published:** 2024-03-25

**Authors:** Pavel V. Chernyshov, Francesca Sampogna, Giulia Raimondi, Christos C. Zouboulis, Michael J. Boffa, Servando E. Marron, Liana Manolache, Nives Pustišek, Vincenzo Bettoli, Dimitra Koumaki, Anthony P. Bewley, Brigitte Dreno, Lucia Tomas-Aragones

**Affiliations:** a Department of Dermatology and Venereology, National Medical University, Kiev, Ukraine; b Clinical Epidemiology Unit, Istituto Dermopatico dell'Immacolata IDI-IRCCS, Rome, Italy; c Department of Developmental and Social Psychology, Sapienza University of Rome, Rome, Italy; d Departments of Dermatology, Venereology, Allergology and Immunology, Staedtisches Klinikum Dessau, Brandenburg Medical School Theodor Fontane and Faculty of Health Sciences Brandenburg, Dessau, Germany; e Department of Dermatology, Mater Dei Hospital, Msida, Malta; f Department of Dermatology, University Hospital Miguel Servet, Aragon Psychodermatology Research Group associated to Aragon Health Sciences Institute (IACS), Zaragoza, Spain; g Dermatology, Dali Medical, Bucharest, Romania; h Children's Hospital Zagreb, Zagreb, Croatia; i Section of Dermatology, Department of Medical Sciences, University of Ferrara, Ferrara, Italy; j Department of Dermatology and Venereology, University Hospital of Heraklion, Heraklion, Greece; k Barts Health NHS Trust, London, UK; l Queen Mary University, London, UK; m Department of Dermato-Oncology, Nantes Université, Univ Angers, INSERM, Immunology and New Concepts in ImmunoTherapy (INCIT), Nantes, France; n Department of Psychology, University of Zaragoza, Aragon Health Sciences Institute (IACS), Zaragoza, Spain

**Keywords:** acne, patient-reported outcome measures, quality of life

## Abstract

**Background:**

Participating members of the European Academy of Dermatology and Venereology Task Forces on quality of life (QoL) and Patient Oriented Outcomes and Acne, Rosacea and Hidradenitis Suppurativa initiated data collection in 9 European countries and formed the list of the most relevant topics for acne patients.

**Objective:**

The aim of this study was to develop a new acne-specific health-related QoL instrument based on the list of the most relevant topics for acne patients.

**Methods:**

After assessment by acne patients (*n* = 715) on how clear and relevant the items in the prototype questionnaire were, a group of experts on acne and QoL performed discussions on items inclusion, which resulted in a series of 21 items. Then another group of acne patients (*n* = 1502) filled in the new version of the instrument. A factor analysis was conducted on the 21-item version.

**Results:**

Three-factor model with 19 items indicated a satisfactory fit. The three dimensions were called: Socioemotional; Symptoms; Stigma and Suicidal thoughts.

**Limitations:**

Included patients and experts may not fully represent acne patients and health care professionals worldwide.

**Conclusion:**

A final 19-item version of the Quality of Life Relevance-Acne was developed.

**Capsule Summary**

• This article provides information on the development of an acne-specific quality of life questionnaire (Quality of Life Relevance-Acne).
• The final version of the Quality of Life Relevance-Acne consists of 3 dimensions and 19 items.

## Introduction

Acne has a significant negative influence on patients’ health-related quality of life (HRQoL).^1^ HRQoL assessment in patients with acne is recommended by several national and international guidelines including the European Dermatology Forum S3-Guideline for the Treatment of Acne and recommendations of the European Academy of Dermatology and Venereology Task Forces on QoL and Patient Oriented Outcomes and Acne, Rosacea and Hidradenitis Suppurativa.^1^^,^^2^ Several different acne-specific instruments are available to assess QoL in patients with acne vulgaris, each one evaluating some QoL domains but none of which address them all.^3^ Participating members of the European Academy of Dermatology and Venereology Task Forces on QoL and Patient Oriented Outcomes and Acne, Rosacea and Hidradenitis Suppurativa initiated data collection in 9 European countries and formed the list of the most relevant topics for acne patients.^4^ The aim of this study was to develop a new acne-specific HRQoL instrument based on the list of the most relevant topics for acne patients.

## Methods

A combination of the most relevant topics and factor analysis results^4^ was used as a prototype of a new acne-specific instrument. Acne patients were asked how clear and relevant the items are.

Group of experts (consisted of all authors from the authors’ list except G.R.) on acne and QoL performed discussions on items inclusion (eg to exclude duplications), more clear formulation and incorporation of additional items, resulting in a list of 21 items. Separate discussion was organized by the expert group on scoring system for the new acne-specific HRQoL instrument.

After that, another group of acne patients filled in the 21-item version of the new acne-specific QoL instrument.

### Statistical analyses

All the analyses were performed with Mplus 8.3 (Muthén & Muthén, 1998-2017) and the packages Mokken (version 3.0.4) and Psych (version 2.3.6) for R.^5^^,^^6^

The analyses were conducted on the pool of 21 items. The sample was randomly split into 2 subsamples using the SPSS function. In the first sample an exploratory factor analysis (EFA) was performed, and items with factor loadings less than 0.40 were removed. The following indices were used to assess model fit: (1) the Root Mean Square Error of Approximation (RMSEA), with values below 0.05 indicating evidence of absolute fit, values between 0.05 and 0.08 indicating adequacy of the model, and values above or equal to 0.10 indicating poor fit of the model^7^^,^^8^; (2) the Tucker Lewis Index, with values ≥ 0.95 indicating good fit of the model and values of 0.90 and higher an acceptable fit; (3) the Comparative Fit Index, with values ≥ 0.95 indicating good model fit and values of 0.90 and higher an acceptable fit; (4) the Standardized Root Mean square Residual, with values < 0.08 indicating good fit^9^; and (5) the chi-square (χ2) test, with *P* values greater than .05 indicating an adequate fit to the data. However, χ2 is sensitive to sample size, and so *P* values might become significant for large samples.^10^ In the second subsample a confirmatory factor analysis (CFA) was performed to confirm the stability of the factor solution obtained in the previous step. The models from both EFA and CFA were evaluated using the Mean-and Variance-adjusted Weighted Least Square estimator with a polychoric correlation matrix. The adequacy of the CFA model was evaluated with the same fit indices reported above.

While it is true that goodness-of-fit indices (eg, RMSEA) are associated with CFA, they are also used in the context of EFA as well, as reported by literature,^11^^,^^12^ since they can offer valuable insights into model fit, even in the absence of a predetermined factor structure.

Indices of internal consistency (ie, ordinal alpha, Molenaar Sijtsma statistic [MS], and latent class reliability coefficient [LCRC]) were calculated on the whole sample. In order to assess the presence of any floor and/or ceiling effect, mean scores for each factor were calculated and skewness and kurtosis of each dimension were inspected.

Ethical approval was obtained from the local ethical research committee. Informed consent from patients was obtained in all cases.

## Results

Acne patients (*n* = 715) were asked to assess how clear the items formed on the base of combination of the most relevant topics and factor analysis results are and about the impact of the items on their life during last 2 weeks. Response rate was from 99.70% to 87.83%. Mean age of patients was 22.66 ± 4.13 years (age ranges: 12-40 years). Patients’ answers on clarity and impact of the first set of items are presented in Table I.

**Table I:** Patients’ (*n* = 715) answers on clarity and impact of the first set of items

Items of the long version		How clear is the item?	Impact of the item during last 2 weeks
1	I feel like an “outcast” because of my acne	Very clear 68.4% (*n* = 699)	67.9% (*n* = 713)
2	I have difficulties in relationships with my spouse/partner because of my acne	Very clear 88.0% (*n* = 686)	37.2% (*n* = 705)
3	I have difficulties in relationship with my close friends because of my acne	Very clear 90.9% (*n* = 681)	34.3% (*n* = 712)
4	I have difficulties in relationship with my immediate family because of my acne	Very clear 91.6% (*n* = 633)	38.5% (*n* = 712)
5	I feel social withdrawal because of my acne	Very clear 90.1% (*n* = 680)	66.5% (*n* = 710)
6	Acne interferes with my sex life	Very clear 91.9% (*n* = 668)	35.1% (*n* = 700)
7	Acne affects my social or leisure activities	Very clear 93.1% (*n* = 666)	79.1% (*n* = 708)
8	My acne stops me from being close with those I love	Very clear 91.7% (*n* = 662)	49.4% (*n* = 702)
9	My acne affects my going out, playing and doing hobbies	Very clear 92.8% (*n* = 667)	57.0% (*n* = 707)
10	I have problems with the use of public changing rooms/swimming pools because of my acne	Very clear 90.6% (*n* = 661)	43.0% (*n* = 703)
11	It was difficult to do any sport because of my acne	Very clear 92.4% (*n* = 662)	37.1% (*n* = 706)
12	I believe that people are staring at me because of my acne	Very clear 95.0% (*n* = 662)	83.6% (*n* = 706)
13	People are calling me names, tease, bully, ask questions or avoid me because of my acne	Very clear 95.0% (*n* = 659)	61.3% (*n* = 703)
14	Acne prevents me from working or studying or I have problems at work or studying	Very clear 94.4% (*n* = 655)	39.1% (*n* = 704)
15	I have sleep problems because of my acne	Very clear 87.6% (*n* = 653)	15.8% (*n* = 703)
16	Acne interferes with my going shopping or looking after my home or garden	Very clear 82.1% (*n* = 653)	35.9% (*n* = 702)
17	I am aggressive because of my acne	Very clear 92.0% (*n* = 649)	79.3% (*n* = 700)
18	I am frustrated because of my acne	Very clear 91.2% (*n* = 649)	96.7% (*n* = 698)
19	I am embarrassed because of my acne	Very clear 90.8% (*n* = 649)	82.5% (*n* = 698)
20	I am worrying or anxious because of my acne	Very clear 92.9% (*n* = 650)	82.9% (*n* = 700)
21	I feel sad because of my acne	Very clear 93.2% (*n* = 648)	95.6% (*n* = 698)
22	I am discouraged because of my acne	Very clear 88.6% (*n* = 643)	82.3% (*n* = 693)
23	I am ashamed because of my acne	Very clear 92.2% (*n* = 640)	79.7% (*n* = 696)
24	I am annoyed because of my acne	Very clear 93.3% (*n* = 638)	76.6% (*n* = 696)
25	I am more irritable because of my acne	Very clear 88.2% (*n* = 642)	70.1% (*n* = 692)
26	I am concerned or worried about meeting new people because of my acne	Very clear 95.3% (*n* = 639)	88.3% (*n* = 693)
27	I tend to do things by myself because of my acne	Very clear 82.6% (*n* = 638)	48.9% (*n* = 687)
28	My acne causes discomfort or pain	Very clear 92.4% (*n* = 633)	83.7% (*n* = 689)
29	My skin feels dirty because of my acne	Very clear 92.0% (*n* = 635)	92.0% (*n* = 688)
30	My skin hurts/is sore because of my acne	Very clear 90.4% (*n* = 633)	96.8% (*n* = 687)
31	My skin burns because of my acne	Very clear 93.0% (*n* = 631)	54.7% (*n* = 688)
32	My skin itches because of my acne	Very clear 95.3% (*n* = 633)	71.9% (*n* = 688)
33	My skin is irritated because of my acne	Very clear 93.9% (*n* = 636)	92.1% (*n* = 687)
34	My skin is sensitive because of my acne	Very clear 95.9% (*n* = 632)	88.0% (*n* = 689)
35	My skin bleeds because of my acne	Very clear 86.4% (*n* = 631)	54.9% (*n* = 680)
36	I am concerned medication will not clear my face acne fast enough	Very clear 91.7% (*n* = 628)	84.1% (*n* = 685)
37	I am annoyed at having to spend time cleaning/treating facial acne	Very clear 94.6% (*n* = 629)	76.7% (*n* = 687)
38	I am concerned about the side effects from treatment of my acne	Very clear 94.6% (*n* = 631)	71.2% (*n* = 681)
39	Water bothers my acne	Very clear 88.8% (*n* = 632)	37.8% (*n* = 682)
40	I am dissatisfied with my self-appearance because of my acne	Very clear 96.4% (*n* = 631)	96.6% (*n* = 686)
41	I wish I looked better because of my acne	Very clear 94.1% (*n* = 632)	100% (*n* = 689)
42	I don’t like photographs of me because of my acne	Very clear 96.5% (*n* = 632)	94.5% (*n* = 688)
43	I am upset about having facial acne	Very clear 95.7% (*n* = 633)	99.0% (*n* = 687)
44	I worry that my acne may get worse	Very clear 96.2% (*n* = 630)	97.7% (*n* = 687)
45	I feel lacking in self-confidence because of my acne	Very clear 95.8% (*n* = 637)	95.1% (*n* = 690)
46	I need to use makeup because of my acne	Very clear 92.1% (*n* = 633)	79.3% (*n* = 691)

Based on 5 rounds of experts group discussions, 29 items from the initial list were deleted or replaced by the following 4 items: “I found it difficult to communicate with others because of my acne”, “I was upset about having acne”, “I have been stressed because of my acne”, “I felt lonely because of my acne”. Meanwhile, 2 new items were added: “My acne caused suicidal thoughts” and “People offered advice, or said things to me about my acne when I had not asked them to do that”. A four-point Likert scale (never, rarely, sometimes, frequently) was selected by the group of experts as a scoring system of the new acne-specific HRQoL questionnaire.

**Fig 1:**
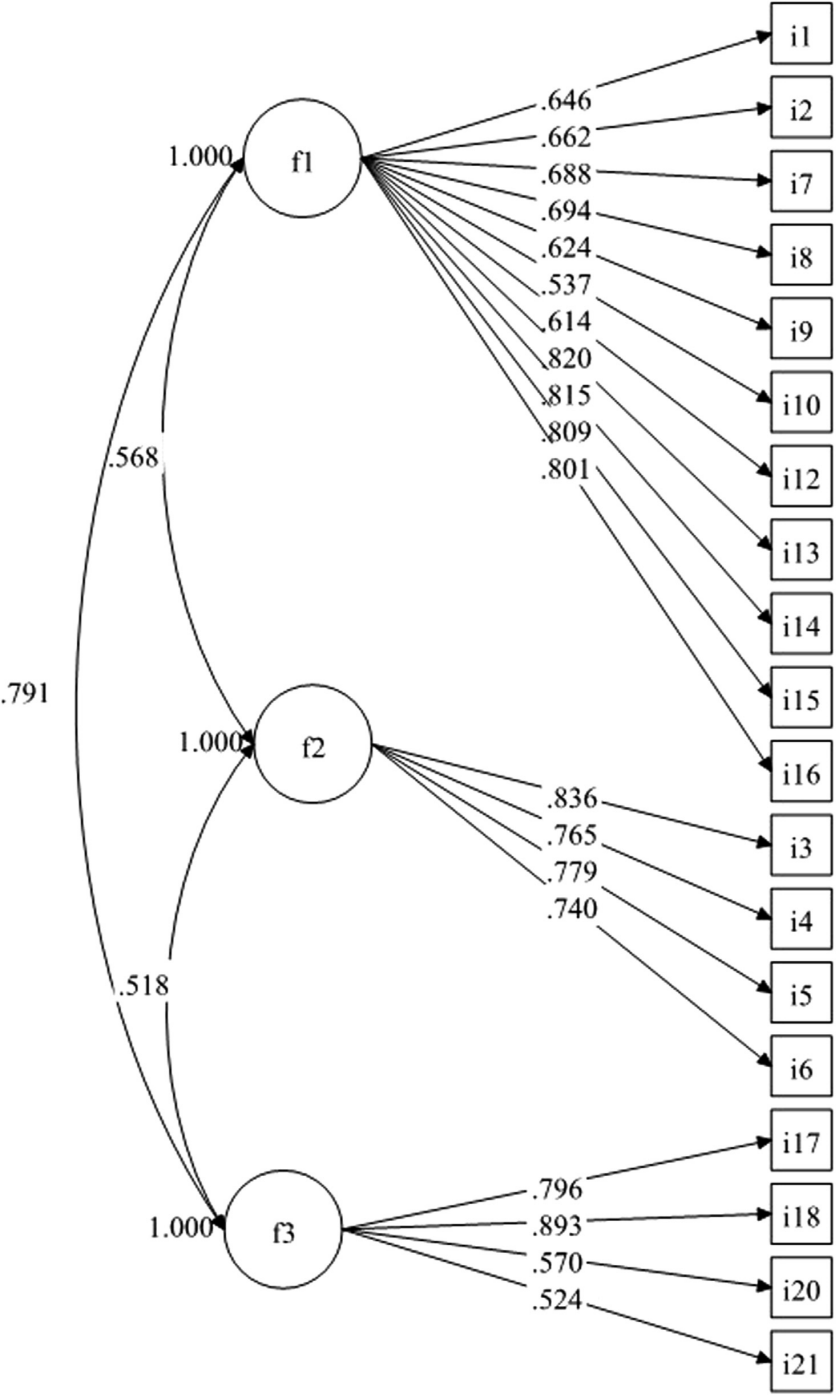
Standardized factor loadings of each dimension. F1 = Socioemotional dimension; F2 = Symptoms dimension; F3 = Stigma and Suicidal thoughts dimension.

Factor analyses were conducted on the final pool of 21 items. Response rate in a second group of patients (*n* = 1502) was 99.73%. Mean age of patients was 24.27 ± 4.72 years (age ranges: 13-41 years). On the first subsample (*n* = 722), the EFA suggested a three-factor model that fitted the data best [χ2 (150) = 511.11, *P* < .001; RMSEA = 0.058, 90% CI = 0.052-0.063; Comparative Fit Index= 0.97; Tucker Lewis Index= 0.96; Standardized Root Mean square Residual = 0.04]. When inspecting the model, results indicated 1 item with low factor loading (<0.40) and 1 item with cross-loadings on 2 factors, therefore these 2 items (“I have been concerned about the side effects from treatment of my acne” and “I felt lonely because of my acne”) were removed from further analyses. The CFA on the second subsample (*n* = 776) of the three-factor model with 19 items indicated a satisfactory fit [χ2 (149) = 664.78, *P* < .001; RMSEA = 0.067, 90% CI = 0.062-0.072; Comparative Fit Index = 0.95; Tucker Lewis Index= 0.94; Standardized Root Mean square Residual = 0.05] (see Fig 1 for standardized factor loadings).

The first dimension included 11 items concerning psychosocial aspects of the disease, such as dissatisfaction with appearance, difficulties in social life, and emotions such as concern about the side effect of the medication, worry, stress, and lack in self-confidence, and being upset. This dimension has been called “Socioemotional”. The second dimension was called “Symptoms”, as it included 4 symptoms. The third dimension included 4 items, 1 of them on suicide and the others on stigma. It was thus called “Stigma and Suicidal thoughts”.

The questionnaire (Table II) showed satisfactory internal consistency for all 3 subscales (Socioemotional: Ordinal alpha = 0.91, MS = 0.88, and LCRC = 0.89; Symptoms: Ordinal alpha = 0.85, MS = 0.81, and LCRC = 0.78; Stigma and Suicidal thoughts: Ordinal alpha = 0.80, MS = 0.73, and LCRC = 0.71). Skewness and kurtosis for the 3 factors were: F1: Skewness = 0.43, Kurtosis = −0.77; F2: Skewness = 1.69, Kurtosis = 2.00; F3: Skewness = 0.81, Kurtosis = 0.28.

**Table II:** Final version of the Quality Of Life Relevance-Acne questionnaire

Quality of Life Relevance-Acne		
1	I have been dissatisfied with my self-appearance because of my acne	1. Frequently
		2. Sometimes
		3. Rarely
		4. Never
2	I have not liked photographs of me because of my acne	1. Frequently
		2. Sometimes
		3. Rarely
		4. Never
3	My skin has burned because of my acne	1. Frequently
		2. Sometimes
		3. Rarely
		4. Never
4	My skin had been itchy because of my acne	1. Frequently
		2. Sometimes
		3. Rarely
		4. Never
5	My skin has been irritated because of my acne	1. Frequently
		2. Sometimes
		3. Rarely
		4. Never
6	My skin has been sensitive because of my acne	1. Frequently
		2. Sometimes
		3. Rarely
		4. Never
7	It was difficult to do sport or use swimming pools or public changing rooms because of my acne	1. Frequently
		2. Sometimes
		3. Rarely
		4. Never
8	Acne has interfered with my sex life	1. Frequently
		2. Sometimes
		3. Rarely
		4. Never or not relevant for me
9	I have been concerned medication will not clear my acne fast enough	1. Frequently
		2. Sometimes
		3. Rarely
		4. Never
10	I have been concerned about the side effects from treatment of my acne	1. Frequently
		2. Sometimes
		3. Rarely
		4. Never
11	I worried that my acne may get worse	1. Frequently
		2. Sometimes
		3. Rarely
		4. Never
12	I found it difficult to communicate with others because of my acne	1. Frequently
		2. Sometimes
		3. Rarely
		4. Never
13	I felt lacking in self-confidence because of my acne	1. Frequently
		2. Sometimes
		3. Rarely
		4. Never
14	I was upset about having acne	1. Frequently
		2. Sometimes
		3. Rarely
		4. Never
15	I have been stressed because of my acne	1. Frequently
		2. Sometimes
		3. Rarely
		4. Never
16	My acne caused suicidal thoughts	1. Frequently
		2. Sometimes
		3. Rarely
		4. Never
17	I felt lonely because of my acne	1. Frequently
		2. Sometimes
		3. Rarely
		4. Never
18	People bullied me because of my acne	1. Frequently
		2. Sometimes
		3. Rarely
		4. Never
19	People offered advice, or said things to me about my acne when I had not asked them to do that	1. Frequently
		2. Sometimes
		3. Rarely
		4. Never

## Discussion

The aim of the current work was to develop a new self-reported acne-specific questionnaire measuring HRQoL based on the most relevant topics for acne patient with additions of items that aimed to identify patients who need immediate psychological help. Our findings suggest the presence of 3 factors, which were named as Socioemotional, Symptoms, and Stigma and Suicidal thoughts. For each factor, skewness and kurtosis fell in the ranges commonly used to assess the normality of the distribution (ie, −2.00 to +2.00).^13^ Therefore, no ceiling or floor effect were detected in our analyses.

It is well-known that acne produces cosmetic disfigurement and patients suffering from visible skin conditions have an increased risk of depression, anxiety, body dysmorphic disorder, feelings of stigmatization, and self-harm ideation.^14^ Feelings about the appearance of the skin over the last month had significant impact on school and university students with acne.^15^ Due to the strong desire to have a clear skin, they might feel the need to constantly hide behind makeup, which further diminishes their confidence.^16^

Severe acne can cause soreness, swelling, and bleeding, making pain and discomfort challenging symptoms of this condition.^17^^,^^18^

Acne can be associated with multiple breakouts, treatment failure, and scarring. The social stigma associated with it can be destabilizing for the individuals, leading to symptoms of anxiety and depression.^19^ In 1 study nearly 2 in 3 teenagers acknowledged that acne had been a source of embarrassment.^20^ Moreover, the social stigma associated with acne, together with the fear of being treated differently, can often lead individuals to withdraw from social interactions, deepening feelings of loneliness and sadness.^21^ The prevalence of bullying, unsolicited advice, and tactless questions among acne patients is high and often lead to long-term negative effects.^22^ Some patients even considered COVID-19 related quarantine as a positive factor to decrease stress, stigmatization, and bullying.^22^^,^^23^

The constant worry that acne can exacerbate these symptoms, making individuals susceptible to a wider range of psychological problems including suicidal thoughts.^24^^,^^25^ Although psychiatric comorbidities frequently accompany acne patients in dermatology, they are almost never directed to dermatology-psychiatry liaison clinics. Depression, anxiety, stress, decreased self-esteem, suicidal thoughts, and even suicide attempts are too frequent to ignore in these patients.^26^ Acne can play an important negative role in sexuality and satisfaction.^27^ Surprisingly, acne’s effect on sexual difficulties, work or school was not associated with the decision to visit a dermatologist in 1 study.^28^

The limitation of our study is that included patients and experts may not be fully representative of all acne patients and health care professionals worldwide.

Most of the items from the initial list that represented topics from acne-specific and dermatology-specific instruments were not relevant to acne patients, especially for more recent time periods.^3^^,^^4^ Therefore, it could be that clinically effective treatment may not apparently improve patients’ quimp during the selected time period. The acne-specific QoL questionnaire Quality of Life Relevance-Acne may help to present detailed analysis of patients’ quimp and therefore may be important to establish individual long-term treatment strategy, to increase treatment adherence, and could be helpful for other dermatological purposes where the QoL measurement is important.^4^^,^^29^ We hope that our new acne-specific HRQoL questionnaire contains the most important and relevant items for acne patients worldwide. A promising and innovative approach is to create new QoL instruments by international groups of specialists, rather than within individual countries, as in the case of the European KIDSCREEN/DISABKIDS project^30^ or the InToDermQoL questionnaire and its epidermolysis bullosa-specific module.^31^^,^^32^ Relevance of topics for acne patient was checked in different European countries and validation of the new acne-specific instrument will be organized simultaneously in different countries and languages to avoid potential cross-cultural inequivalence.

The methodology of QoL instrument validation is constantly becoming more rigorous and practically oriented.^33^ In order to define when the change in a score of a HRQoL instrument becomes ‘significant’ to a patient, the minimal clinically important difference (MCID) can be calculated. The MCID represents the smallest improvement considered worthwhile by a patient. The concept of an MCID is offered as the new standard for determining minimal effectiveness of a given treatment and for describing patient satisfaction in reference to that treatment.^34^ In order to give clinically useful meaning to QoL scores, it is possible to define score band descriptors.^35^ Detailed recommendations on treatment goals and changes of treatment approaches, based on a HRQoL questionnaire with a validated banding system, may be an important and promising approach.^3^ MCID and score banding system were established for the most widely used dermatology-specific HRQoL instrument the Dermatology Life Quality Index and for the InToDermQoL questionnaire.^36^, ^37^, ^38^ Therefore extensive validation and establishing severity banding system and MCID for the Quality of Life Relevance-Acne questionnaire is an important task for future studies. QoL assessment in dermatology is a rapidly developing field with a gradual shift from theory to practice^33^ and we hope that our new questionnaire will be used not only in research studies and core outcome sets^39^ but also in clinical practice.

## Conflicts of interest

None declared.
